# Dysregulation of Angiopoietins Is Associated with Placental Malaria and Low Birth Weight

**DOI:** 10.1371/journal.pone.0009481

**Published:** 2010-03-01

**Authors:** Karlee L. Silver, Kathleen Zhong, Rose G. F. Leke, Diane Wallace Taylor, Kevin C. Kain

**Affiliations:** 1 McLaughlin-Rotman Centre for Global Health, McLaughlin Centre for Molecular Medicine, University Health Network, University of Toronto, Toronto, Ontario, Canada; 2 Faculty of Medicine and Biomedical Sciences, University of Yaoundé 1, Yaoundé, Cameroon; 3 Department of Biology, Georgetown University, Washington, D.C., United States of America; 4 Department of Tropical Medicine, John A. Burns School of Medicine, University of Hawaii, Honolulu, Hawaii, United States of America; 5 Tropical Disease Unit, Division of Infectious Diseases, Department of Medicine, Toronto General Hospital, University of Toronto, Toronto, Ontario, Canada; Walter and Eliza Hall Institute of Medical Research, Australia

## Abstract

**Background:**

Placental malaria (PM) is associated with adverse pregnancy outcomes including low birth weight (LBW). However, the precise mechanisms by which PM induces LBW are poorly defined. Based on the essential role of angiopoietin (ANG)-1 and -2 in normal placental vascular development, we hypothesized that PM may result in the dysregulation of angiopoietins and thereby contribute to LBW outcomes.

**Methods and Findings:**

In a mouse model of PM, we show that *Plasmodium berghei* ANKA infection of pregnant mice resulted in dysregulated angiopoietin levels and fetal growth restriction. PM lead to decreased ANG-1, increased ANG-2, and an elevated ratio of ANG-2/ANG-1 in the placenta and the serum. These observations were extended to malaria-exposed pregnant women: In a study of primigravid women prospectively followed over the course of pregnancy, *Plasmodium falciparum* infection was associated with a decrease in maternal plasma ANG-1 levels (*P* = 0.031) and an increase in the ANG-2:ANG-1 ratio (*P* = 0.048). ANG-1 levels recovered with successful treatment of peripheral parasitemia (*P* = 0.010). In a cross-sectional study of primigravidae at delivery, angiopoietin dysregulation was associated with PM (*P* = 0.002) and LBW (*P* = 0.041). Women with PM who delivered LBW infants had increased ANG-2:ANG-1 ratios (*P* = 0.002) compared to uninfected women delivering normal birth weight infants.

**Conclusions:**

These data support the hypothesis that dysregulation of angiopoietins is associated with PM and LBW outcomes, and suggest that ANG-1 and ANG-2 levels may be clinically informative biomarkers to identify *P. falciparum*-infected mothers at risk of LBW deliveries.

## Introduction

Low birth weight (LBW) infants have increased rates of mortality during the first year of life [1], [2]. Placental malaria (PM) due to *Plasmodium falciparum* infection doubles the risk of LBW, and results in an estimated 100 000 infant deaths per year [3]. Despite a clear association of LBW with PM and high mortality rates, the mechanisms by which malaria infection of the placenta induces LBW are poorly defined.

PM-associated LBW is a result of intrauterine growth restriction (IUGR) and/or premature delivery (<37 weeks of gestation) [4]. Both IUGR and spontaneous preterm delivery can result from functional placental insufficiency [5]-[8] where nutrient supply is inadequate to support fetal growth and continued in utero development. A key factor associated with placental insufficiency is the abnormal formation of the vascular network within the placenta [9], [10]. A complex interplay of angiogenic factors is required for formation of normal placental villous vasculature.

Angiopoietins (ANG-1 and ANG-2) are critical regulators of vascular development and angiogenesis. The placental and systemic expression levels of ANG-1 and ANG-2 in normal and pathological pregnancy has been explored in numerous studies [11]–[20]. In situ hybridization and immunohistochemical studies of human placentas have identified the syncytiotrophoblast and cytotrophoblasts as sources of ANG-1 and ANG-2, and placental macrophages and endothelial cells as sources of ANG-2 [11], [13], [15], [18], [19], [21]. ANG-1 promotes vascular maturation while ANG-2 destabilizes the vasculature and promotes angiogenesis. In concert with vascular endothelial growth factor (VEGF), the angiopoietins have been proposed to guide fetal trophoblast invasion of the uterine wall and spiral artery remodeling at mid-gestation (16–20 weeks), as well as drive the continuous vascular remodeling required to sustain fetal growth in the third trimester [18], [20]. Abnormal expression of other angiogenic factors, VEGF and its soluble receptor sFlt1, has been associated with PM in first time mothers at delivery [22].

After birth, the angiopoietin system regulates the integrity of the vascular endothelium. The constitutive interaction of ANG-1 with Tie-2 maintains the integrity and quiescent nature of the mature vascular enthothelium [23], [24]. As ANG-2 is an antagonist of ANG-1/Tie-2 interactions, its expression is under tight regulation. Both TNF and VEGF are able to induce endothelial cell ANG-2 transcription and de novo protein expression [25], [26]. ANG-2 is also stored in endothelial intracellular vesicles, termed Weibel-Palade bodies, that can be rapidly released upon endothelial activation [27]. Recently, increased circulating ANG-2 levels were associated with severe and cerebral malaria in three distinct populations [28]–[30], while ANG-1 levels and the ratio of ANG-2/ANG-1 were shown to differentiate between individuals with cerebral malaria and those with uncomplicated malaria, in addition to predicting mortality in Ugandan children with cerebral malaria [29].

Based on the observations that angiopoietins play an important role in pregnancy and are dysregulated with severe and cerebral malaria, we hypothesized that angiopoietin dysregulation also occurs with PM, and that this contributes to LBW outcomes associated with PM. To test this hypothesis, we examined angiopoietin levels in an experimental PM mouse model and in malaria-exposed pregnant women. We report that altered angiopoietin levels are associated with PM and LBW.

## Materials and Methods

### Ethics Statement

Studies of pregnant women were approved by the Institutional Review Board, Georgetown University; the National Ethical Committee, Ministry of Public Health, Cameroon; and the National Institutes of Health. Written or verbal consent was obtained from each study participant: Written informed consent was obtained when women were able to read the consent form, otherwise documented verbal consent was obtained (i.e., a third party (usually a friend of the participant) signed that the woman had consented). This consent approach was specifically approved by all review bodies due to the high number of illiterate women in the study region.

Mouse experiments were approved by the University Health Network Animal Care Committee and performed in accordance with current institutional and national regulations, including the Canadian Council on Animal Care's *Guide to the Care and Use of Experimental Animals* and the Ontario Society for the Prevention of Cruelty to Animals Act. Mice were maintained on a 12-hr dark and 12-hr light cycle with free access to feed and water.

### Murine Placental Malaria Model

Eight to ten week-old BALB/c mice were obtained from Jackson Laboratories (Bar Harbor, ME). Experimental PM was induced by infecting pregnant BALB/c females with *Plasmodium berghei* ANKA as previously described [31]. Briefly, female mice were mated with males, and checked daily for the presence of a vaginal plug (gestational day 1 (G1)). Cryopreserved *P. berghei* ANKA (MR4; Manassas, VA) was thawed and passaged through male BALB/c mice. Upon confirmation of pregnancy by observation of 3–4 g body weight increase, pregnant females were infected on G13 with 10^6^ *P. berghei*-infected erythrocytes in RPMI media via injection into the lateral tail vein. Control pregnant females were injected with the same volume of RPMI media alone.

Parasitemia was monitored daily by thin blood smear stained with modified Giemsa stain (Protocol Hema3 Stain Set, Sigma, Oakville, ON). Pregnant female mice were euthanized by CO_2_ on days 16, 18 or 19 post-conception, (i.e., days 3, 5 or 6 post infection/control injection). Blood was collected by cardiac puncture, centrifuged (13,000 rpm for 5 minutes), and serum stored at −80°C until analyzed. Uteri were removed and examined for evidence of resorptions (necrotic bodies and scarring). Yolk sacs were dissected from uteri, fetuses were removed and weighed, and placentas were snap frozen and stored at −80°C until analyzed. Fetal viability was determined by assessing pedal withdrawal reflex. Non-viable fetuses (i.e., lacking the pedal withdrawal reflex) were considered aborted.

### Placenta qRT-PCR for ANG-1 and ANG-2

RNA was extracted from snap-frozen placentas after homogenization in TRIzol (1 mL/100 mg tissue; Invitrogen, Burlington, ON) according to manufacturer's protocol. Extracted RNA (2 µg per sample) was treated with DNase I (Ambion, Streetsville, ON) and reverse transcribed to cDNA with SuperScript III (Invitrogen, Burlington, ON) in the presence of oligo(dT)_18_ primers (Fermentas, Burlington, ON). Residual RNA was degraded with RNase H (Invitrogen, Burlington, ON). Sample cDNA was amplified in triplicate with SYBR Green master mix (Roche, Laval, QC) in the presence of 1 µM both forward and reverse primers in a Light Cycler 480 (Roche, Laval, QC). Transcript number was calculated based on Ct as compared to a standard curve of mouse genomic DNA included on each plate by Light Cycler 480 software (Roche, Laval, QC), and normalized by geometric averaging of GAPDH and HPRT expression levels as previously described [32]. Primer sequences (5′–3′): ANGPT1, *F*- cctctggtgaatattggcttggga , *R*- agcatgtactgcctctgactggtt ; ANGPT2, *F*- agagtactggctgggcaatgagtt , *R*- ttcccagtccttcagctggatctt ; GAPDH, *F*- tcaacagcaactcccactcttcca , *R*- ttgtcattgagagcaatgccagcc ; HPRT, *F*- ggagtcctgttgatgttgccagta , *R*- gggacgcagcaactgacatttcta .

### Placenta Western Blots

Snap-frozen placentas were homogenized in RIPA buffer [150 mM NaCl, 1% NP40, 0.5% deoxycholate, 0.1% SDS, 50 mM TRIS-HCl, pH 8.0, containing complete protease inhibitor cocktail (Roche, Laval, QC)] at a ratio of 10 µL buffer/mg tissue. Homogenates were incubated on ice for 30 minutes then centrifuged (10,000×*g*, 10 minutes, 4°C) twice, transferring the supernatant to a fresh tube between centrifugation steps.

Placental lysate protein (10 µg) was heated to 100°C for 5 minutes in denaturing loading buffer, centrifuged (10,000×*g*, 5 minutes), electrophoresed on 10% SDS-PAGE gel, then transferred to PVDF membrane. Membranes were blocked [Tris buffered saline (TBS) containing 0.05% (v/v) Tween-20 and 5% (w/v) milk powder] for 1 hour at room temperature. Membranes were incubated overnight at 4°C in blocking buffer containing mouse anti-β actin (AC-40, 1∶10,000; Sigma, Oakville, ON) and rabbit polyclonal anti-angiopoietin-1 (1∶1000; Abcam, Cambridge, MA) or rabbit polyclonal anti-angiopoietin-2 (1∶500; ADI, San Antonio, TX). Membranes were washed [TBS containing 0.05% (v/v) Tween-20] for 3×10 minutes and incubated with HRP-conjugated anti-rabbit-IgG and anti-mouse-IgG antibodies (BioRad, Mississauga, ON) for 1 hour at room temperature. Signal was developed by a 2-minute incubation with SuperSignal West Picoluminescent substrate (Pierce, Rockford, IL); membranes were exposed to autoradiation film, which was developed with a medical film processor (Konica Minolta, Wayne, NJ). The amount of angiopoietin protein in placentas was quantified by densitometry using Scion Image 4.0.3.2 (Scion, Frederick, MD) and expressed normalized to the density of the actin band.

### Mouse Serum Angiopoietin-1 Measurement

The mouse and human ANG-1 protein sequences are 97% homologous; therefore, we measured mouse serum ANG-1 levels with a human ANG-1 duoset (R&D, Minneapolis, MN). A titration of recombinant peptide corresponding to amino acids 20-128 of mouse ANG-1 (Primm, Cambridge, MA) was used to gauge the specificity to mouse ANG-1, and was appropriately detected by the human reagents. Serum ANG-1 concentration was determined by comparison to the human ANG-1 recombinant protein standard curve included on each plate.

### Human Study Participants and Plasma Samples

A cohort of pregnant women was prospectively followed over the course of pregnancy in Yaoundé and Ngali II, Cameroon during 2001–2004 [33]. Peripheral plasma samples were collected, thin and thick blood smears were prepared, and clinical history, including antimalarial use and malaria symptoms, were obtained at up to seven monthly visits per woman spanning all three trimesters of pregnancy. Peripheral parasitemia was determined from thick and thin blood smears of samples stained with Diff-Quik (Baxter Scientific, Miami, FL) and examined for the presence of *P. falciparum*. Women who were blood-smear positive for *P. falciparum* were prescribed antimalarial treatment according to the Cameroon Ministry of Health's policy. In addition, peripheral plasma samples, thin and thick blood smears, intervillous space (IVS) blood and placental impression smears were collected at delivery, and infant weight was recorded. PM was defined as the detection of *P. falciparum* parasites in IVS blood smears and in impression smears of placental tissue.

Samples from all study participants who satisfied the following inclusion criteria were tested: primigravidae with live singleton birth, minimum of three peripheral plasma samples from different gestation points available for testing, and PM infection status determined at delivery. The characteristics of the study participants analyzed for angiopoietin levels are presented in Table 1.

**Table 1:** Characteristics of subjects from prospective study population tested for angiopoietin levels.

	Aparasitemic	Parasitemic	*P*
**Number of participants**	8	15	
**Age (years)** a	22.5±4.4	20.5±4.6	0.243^b^
**Number (%) with placental ** ***P. falciparum*** ** at delivery**	0 (0%)	8 (53%)	0.019^c^
**Gestational age at delivery (weeks)** a	41.0±1.5	38.4±3.2	0.041^d^
**Birth weight (g)** a	3031±446	2843±378	0.293^d^

a Mean ± SD.

b Mann-Whitney test.

c Fisher's exact test.

d Unpaired t-test.

To further evaluate the association of angiopoietin levels with LBW outcomes in a different study design and population, peripheral blood samples and paired placental blood samples collected from the IVS, were selected from a cross-sectional study conducted in Yaoundé, Cameroon during 1995–2001 [34]. Peripheral parasitemia was determined from thick and thin blood smears of samples stained with Diff-Quik (Baxter Scientific, Miami, FL) and examined for the presence of *P. falciparum*. PM was defined as detection of *P. falciparum* parasites in thick and thin blood smears of IVS blood and impression smears of placental tissue. Plasma samples were stored at −80°C.

Samples (n = 177) were selected based on the following criteria: consecutively enrolled primigravid women with PM delivering live singleton neonates with LBW (<2500 g) or normal birth weight (NBW; ≥2500 g). Control groups of consecutive women without PM who delivered live NBW or LBW singleton babies were also included in the study. The characteristics of these study participants are presented in Table 2.

**Table 2:** Characteristics of cross-sectional study participants tested for angiopoietin levels.

	PM- NBW	PM- LBW	PM+ NBW	PM+ LBW
**Number of participants**	47	44	51	35
**Age** (years)	22.2±5.0	21.2±4.7	20.9±4.3	20.4±3.7
**Placental parasitemia** (% of erythrocytes parasitized)	0	0	6.0±13.8	6.5±15.3
**Gestational age at delivery** (wks)	38.9±2.4	35.8±4.0 *	39.2±3.2	34.9±3.8 ** §
**Birth weight** (g)	3166±413	2022±415 **	3120±404	1977±429 ** §
**Number of preterm deliveries**	8 (16%)	26 (59%) ***	10 (20%)	24 (69%) *** ∧

Dunn's multiple comparison test: *P<0.01 cp to PM- NBW;

**P<0.001 cp to PM- NBW;

§*P*<0.05 cp to PM+ NBW;

Fisher's exact test: *** *P*<0.0001 cp to PM- NBW;

^∧^ *P*<0.0001 cp to PM+ NBW.

### Plasma Angiopoietin Level Measurements

Plasma levels of ANG-1 and ANG-2 were measured by enzyme immunoassay using the appropriate antibody pair and standard duosets (R&D, Minneapolis, MN). Plasma samples were diluted 1∶10 (ANG-1) or 1∶5 (ANG-2).

### Statistical Analyses

Two-way ANOVA tests with Bonferonni post-tests or t-tests (unpaired for normally distributed data, and Mann-Whitney for non-normally distributed data) were conducted as appropriate for mouse experiments. A mixed linear multivariate model was applied to the prospective study sample results, with gestational age and presence of peripheral parasites as fixed variables, and study participant as a random variable. Pairwise analysis of angiopoietin levels upon resolution of parasitemia was performed by paired t-test. Cross-sectional study ANG-1 and ANG-2 levels were analyzed by Mann-Whitney test. ANG-2/ANG-1 ratio values were log-transformed to achieve a normal distribution then analyzed by unpaired t-test (with Welch's correction for unequal variances, where applicable). Statistical analyses were performed using Prism 4.03 (GraphPad Software, La Jolla, CA) and/or SPSS (Chicago, IL).

## Results

### Spontaneous Abortion and IUGR in Experimental Placental Malaria Model

**Figure 1:**
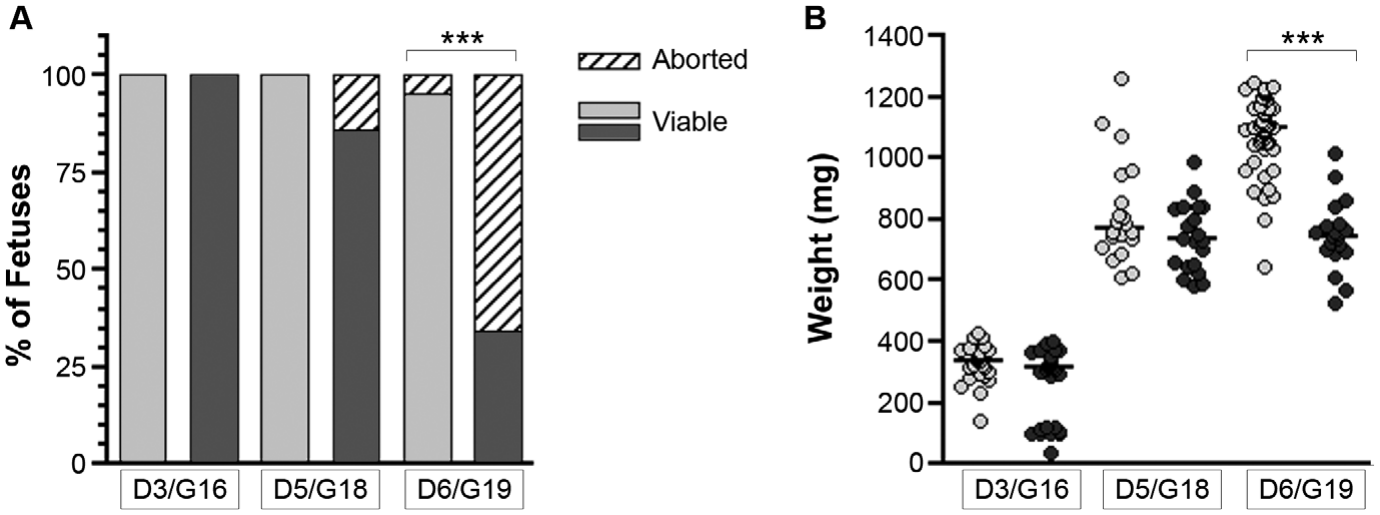
Experimental model recapitulates the spontaneous abortion and low birth weight outcomes characteristic of PM. *(A)* Pregnant BALB/c females infected (dark bars) with *P. berghei* on gestational day (G)13 have an increased rate of abortion and decreased proportion of viable pups per litter compared to uninfected mice (light bars) by G19, day 6 of infection/control injection (D6). Proportion of viable fetuses depicted by solid bars; aborted fetuses, by striped bars. *(B)* Body weight of viable fetuses is decreased with maternal malaria infection (dark symbols) as compared to weight of fetuses from uninfected mice (light symbols). Dots are individual viable fetuses; bars represent the median of each group. 20–83 fetuses were collected per group (from 4–13 pregnant females per group). *** *P*<0.001 (Mann-Whitney).

We used an experimental mouse model to replicate the adverse fetal outcomes of PM in primigravid women [31]. Naturally mated BALB/c females were infected on gestational day (G) 13 via intravenous injection of 10^6^ *P. berghei*-infected erythrocytes obtained from a BALB/c passage mouse. Control pregnant females received an intravenous injection of RPMI media on G13. Litters of injected pregnant females were analyzed at G16 (3 days post injection (D3); n = 5 uninfected and n = 5 infected), at G18 (D5; n = 4 uninfected and n = 5 infected) and at G19 (D6; n = 10 uninfected and n = 13 infected). By G19/D6, the viability rate of fetuses from infected pregnant BALB/c females was only 34±6%, in contrast to 95±15% for fetuses from uninfected controls (*P*<0.001; Fig. 1A). Control values are in agreement with the fetal viability originally reported at G18 for this model [31]. In our hands, the majority of non-resorbed G18 fetuses were viable, regardless of infection status.

Malaria infection also affected the weight of the developing fetuses. At G19, the mean weight of viable fetuses from infected pregnant BALB/c females was lower than that of viable fetuses from uninfected mice (*P*<0.0001; Fig. 1B). In contrast, G18 fetuses from both groups had comparable weights. Fetuses from uninfected mice gained 31±15% extra weight between G18 and G19 (*P*<0.0001), while viable fetuses from infected mice showed no growth over the same period of time (*P* = 0.903; Fig. 1B). Thus, we were able to recapitulate two main adverse birth outcomes associated with PM – spontaneous abortion and IUGR – with this experimental mouse model.

### Angiopoietin Levels Are Altered in Experimental PM

**Figure 2:**
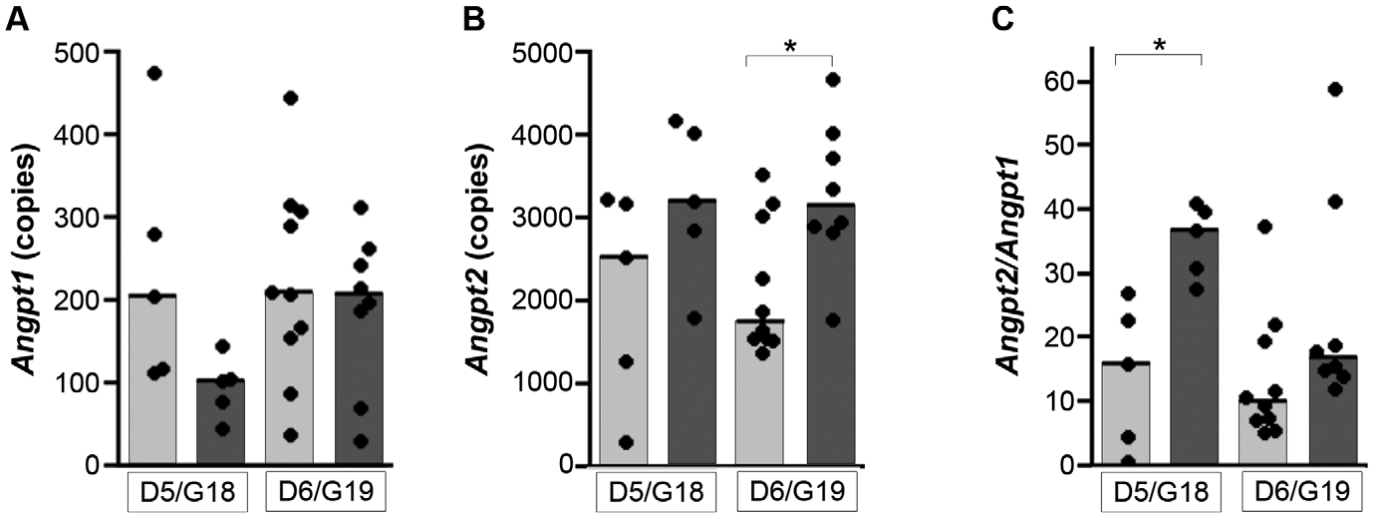
Placental *Angpt2* mRNA expression is increased by malaria infection (*P* = 0.0063, 2-way ANOVA). *(A*) *Angpt1* and *(B*) *Angpt2* transcripts were measured by real-time quantitative PCR using cDNA templates reverse transcribed from placental RNA from pregnant mice uninfected (light bars) and infected with *P. berghei* (dark bars). Copy number was normalized to housekeeping gene expression as described in Materials and Methods. *(C)* The relative expression of *Angpt2*/*Angpt1* is also increased in placentas associated with viable fetuses of infected mice (*P* = 0.0032, 2-way ANOVA on log-transformed data). Dots are individual placentas associated with viable fetuses; bars represent the median of each group. 4–6 mice are represented per group. *P<0.01 (Bonferonni post-test). D, day post infection/control injection; G, gestational day.

In order to test the hypothesis that PM is associated with dysregulation of the angiogenic factors ANG-1 and ANG-2, quantitative real-time PCR (qRT-PCR) of *Angpt1* and *Angpt2* genes was performed on cDNA transcribed from placental RNA. Since we were also interested in the timing of angiogenic dysregulation in relation to the observed fetal growth restriction, we examined placentas of viable fetuses at G18 (i.e., precluding fetal growth restriction, n = 5 placentas from 3 uninfected and 3 infected mice) and G19 (i.e., after onset of growth restriction, n = 10 placentas from 7 uninfected mice, n = 8 placentas from 4 infected mice). Transcription of *Angpt2* and the ratio of *Angpt2/Angpt1* was significantly increased in placentas of infected mice (2-way ANOVA: infection, *P* = 0.0063 and *P* = 0.0031, respectively; Fig. 2), and *Angpt1* showed a trend towards decreased expression in placentas of infected as compared to uninfected mice (2-way ANOVA: infection, *P* = 0.056). Marked angiopoietin ratio dysregulation was observed at G18 (Fig. 2C), prior to the observation of IUGR.

**Figure 3:**
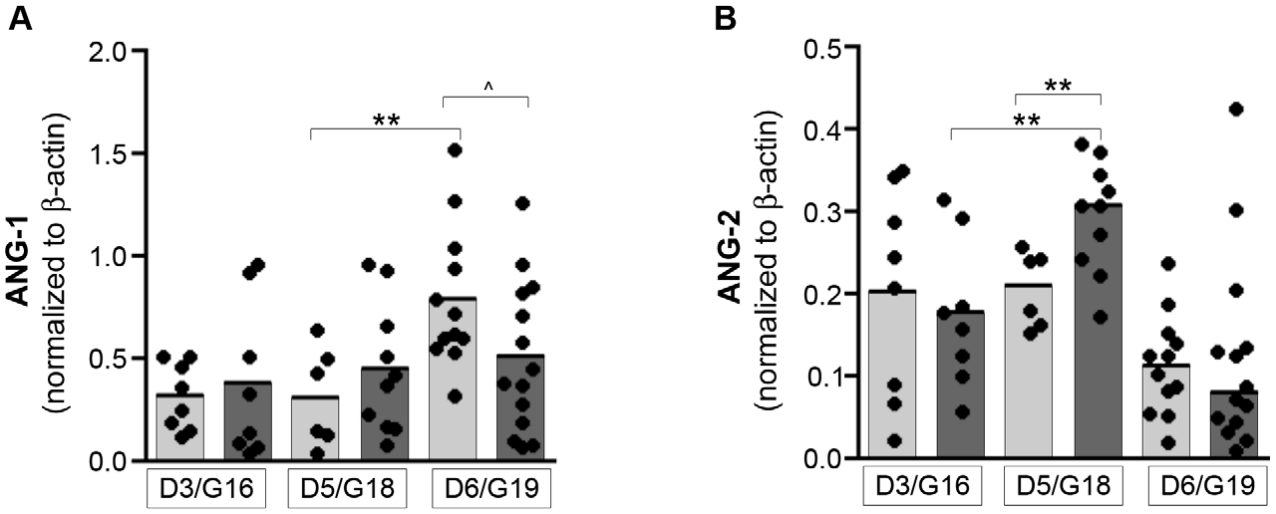
Placental ANG-1 and ANG-2 protein levels are dysregulated by malaria infection. *(A)* ANG-1 and *(B)* ANG-2 protein levels in placentas from pregnant mice uninfected (light bars) and infected with *P. berghei* (dark bars). Protein levels were detected by Western blot, quantified by densitometry and normalized to intensity of β-actin bands. Dots are individual placentas associated with viable fetuses; bars represent the median of each group. 3–13 mice are represented per group. **P<0.01, ∧ *P* = 0.053 (unpaired t-test). D, day post infection/control injection; G, gestational day.

To determine if malaria infection also altered the placental angiopoietin protein levels, semi-quantitative Western blots of lysates from placentas of viable G19 fetuses were performed (Fig. 3; n = 11 placentas from 8 uninfected mice, n = 14 placentas from 6 infected mice). We also examined a random selection of placentas from pregnant mice euthanized at earlier times (G16/D3 n = 8 placentas from 4 uninfected mice, n = 8 placentas from 5 infected mice; G18/D5 n = 6 placentas from 3 uninfected mice, n = 10 placentas from 5 infected mice). Both ANG-1 and ANG-2 levels changed with gestational time, regardless of infection (2-way ANOVA: *P*<0.01 and *P*<0.001, respectively). In the case of ANG-1, the mean level increased with time, which is consistent with reports that *Angpt1* mRNA levels in the placenta increase throughout human pregnancy [18]. An increase in mean uninfected placental ANG-1 expression was observed between G18 and G19 (0.32 to 0.80, *P* = 0.008); however, this increase was absent in the placentas of the infected mice over the same time (0.45 to 0.51, *P* = 0.691; Fig. 3A). At G19, the placentas of infected mice showed a trend towards less ANG-1 than those of uninfected mice (0.51 vs 0.80, *P* = 0.053; Fig. 3A). The mean level of ANG-2 declined with the course of pregnancy, also paralleling what occurs in human pregnancy at the RNA and protein levels [18]. In contrast to the unchanged mean level of ANG-2 in the placentas of uninfected mice from G16 to G18, the mean ANG-2 levels in placentas of infected mice was almost two-fold higher at G18/D5 as compared to G16/D3 (0.29 vs 0.18, *P* = 0.006; Fig. 3B). At G18/D5, the placentas of *P. berghei*-infected pregnant mice had increased mean ANG-2 levels compared those from uninfected mice (0.29 vs 0.21, *P*<0.01, Fig. 3B).

These results show that the placentas of viable but low weight fetuses resulting from experimental PM are associated with dysregulated angiopoietin expression at both transcript and protein levels, and this dysregulation precedes the observed malaria-associated fetal growth restriction. Taken together, these data support the hypothesis that malaria infection-associated angiopoietin dysregulation could play a pathophysiological role in growth restriction.

**Figure 4:**
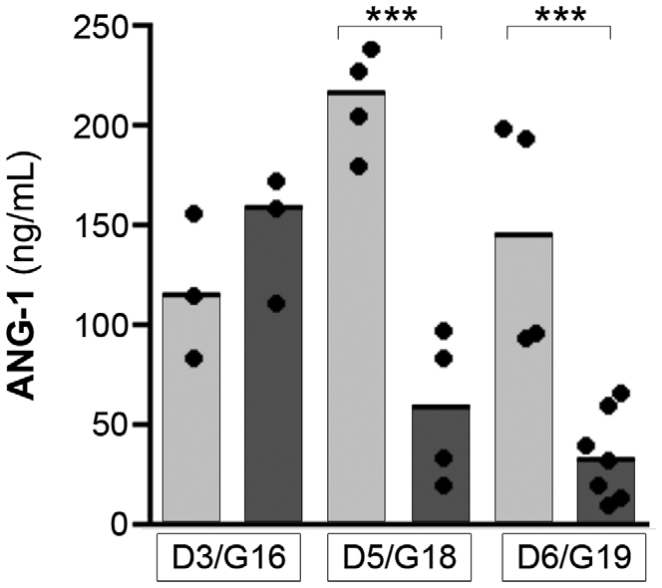
Malaria infection decreases maternal serum ANG-1 in pregnant mice (*P *<0.001, 2-way ANOVA). Serum ANG-1 levels from uninfected (light bars) and *P. berghei* infected (dark bars) pregnant mice as measured by ELISA. Dots are individual mice; bars represent the median of each group. *** *P*<0.001 (Bonferonni post-test). D, day post infection/control injection; G, gestational day.

While information on angiopoietin levels within the placenta provides the most relevant data on pregnancy outcomes, we also investigated whether systemic levels of angiopoietins are altered, as has been reported in humans with cerebral and severe malaria [29], [30]. We used an immunoassay to determine if circulating maternal peripheral blood ANG-1 levels were similarly altered during experimental PM. Serum ANG-1 levels were significantly decreased at G18/D5 and G19/D6 in infected pregnant mice compared to uninfected pregnant mice (2-way ANOVA: (infection × gestational age) interaction, *P*<0.001; Fig. 4). We were unable to measure serum ANG-2 levels in the mice due to an absence of antibodies against native mouse ANG-2.

### Angiopoietin Levels Are Altered in Pregnant Women Infected with *P. falciparum*

**Figure 5:**
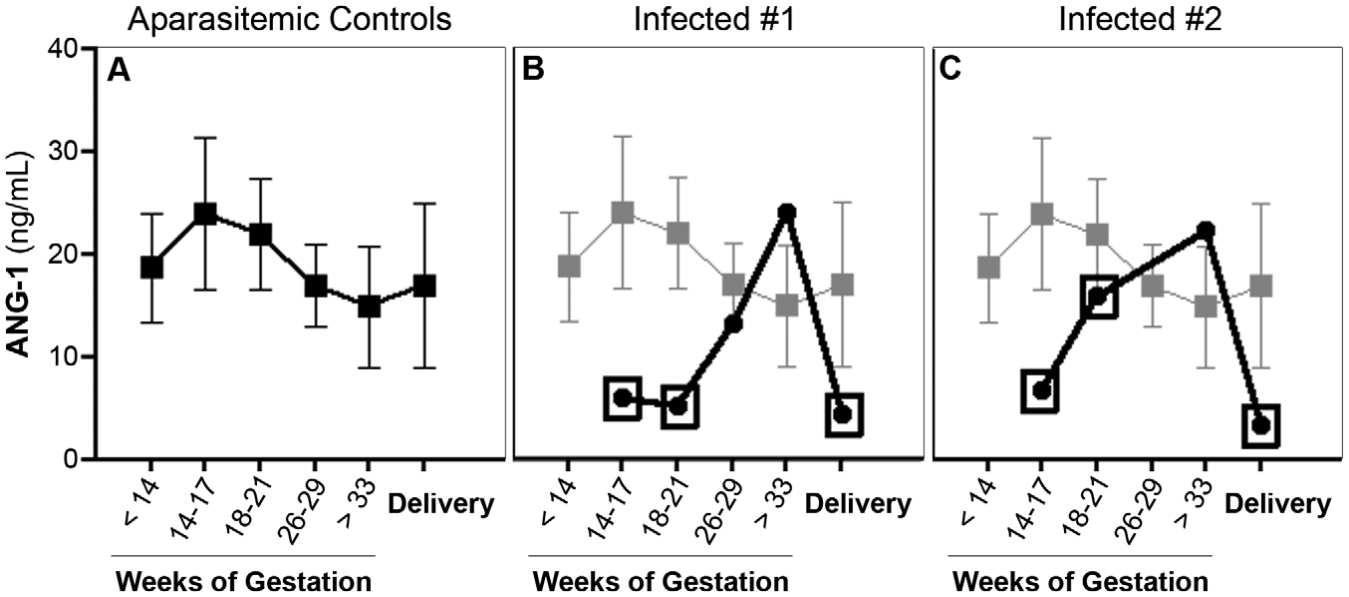
The presence of peripheral parasitemia during pregnancy correlates with decreased plasma ANG-1 levels (*P* = 0.031, mixed linear model). *(A)* Peripheral plasma ANG-1 levels (± SEM) for uninfected primigravid women who had no detectable peripheral or placental parasitemia during the course of the study (n = 8). *(B & C)* Peripheral plasma ANG-1 levels from two representative primigravid women with PM. The mean levels for uninfected women are shown as reference (in light shade). Boxed data points represent visits where women were peripheral blood-smear positive for *P. falciparum*.

We next extended our observations from the mouse model to malaria-exposed pregnant women. We measured ANG-1 and ANG-2 levels in peripheral plasma of 23 primigravid women who were followed prospectively throughout gestation (Fig. 5). 15 of the 23 (62.3%) women were blood-smear positive for *P. falciparum* at least once over the course of pregnancy (Table 1). ANG-1 levels were significantly decreased in the plasma of women when they were blood-smear positive compared to when they were blood-smear negative (Fig. 5B, C vs 5A). When we accounted for the gestational age with a multivariate analysis, presence of peripheral parasitemia was associated with a decrease of 7.3 ng/mL in mean plasma ANG-1 levels (*P* = 0.031). The mean plasma level in aparasitemic pregnant women was 18.8±14.4 ng/mL.

Likewise, infection with *P. falciparum* increased the ANG-2:ANG-1 ratio by 0.328 units (*P* = 0.048). The mean plasma ANG-2:ANG-1 ratio in aparasitemic women was 0.426±0.588. We observed a decline in plasma ANG-2 levels in the last two trimesters (≥14 weeks gestation), as has been previously reported [16]; *P. falciparum* infection did not appear to significantly alter this trend (*P* = 0.434).

**Figure 6:**
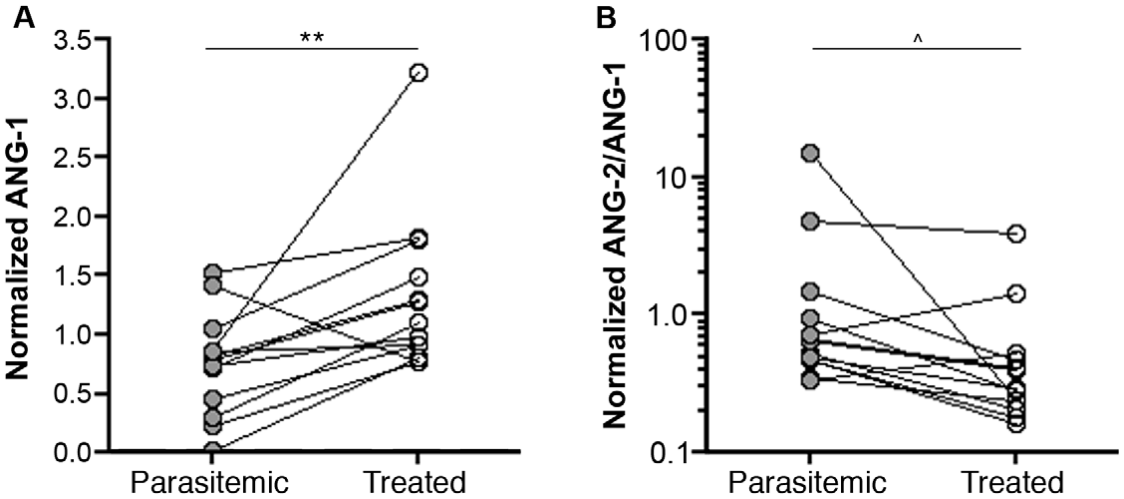
Angiopoietin-1 levels are increased upon resolution of peripheral parasitemia. Paired peripheral plasma *(A)* ANG-1 and *(B)* ANG-2/ANG-1 levels of primigravid women at consecutive visits: the first, when parasitemic by peripheral blood smear microscopy, and the next, when successfully treated and blood-smear negative. To account for physiological variation in angiopoietin levels with gestational age, values were normalized to mean value of aparasitemic controls at the corresponding gestational age. n = 13 pairs. ** *P*<0.01, ^∧^ *P*>0.05 (paired t-test).

Women with detected peripheral parasitemia were prescribed antimalarial chemotherapy, and several resolved their peripheral infection by the following visit. These events occurred at different gestational ages in different individuals. Pairwise analysis of normalized peripheral plasma angiopoietin levels at these two points (parasitemic followed by aparasitemic) revealed a significant increase in normalized ANG-1 levels with parasite clearance regardless of when parasitemia occurred in gestation (*P* = 0.010; Fig. 6A). A trend towards decreased ANG-2/ANG-1 upon resolution of peripheral parasitemia was also observed; however, it did not reach statistical significance (*P* = 0.232; Fig. 6B). These results show that *P. falciparum* infection of pregnant women is associated with systemic, and reversible, angiopoietin dysregulation.

Since only a small number of women (n = 2) in this study delivered LBW babies, it was not possible to determine if the observed angiopoietin dysregulation was also associated with LBW outcomes.

### Angiopoietin Dysregulation at Delivery Is Associated with PM and LBW

To extend our observations to a different population and study design, and to further explore the hypothesis that angiopoietin dysregulation is associated with the LBW outcome observed in PM, we tested the plasma ANG-1 and ANG-2 levels from primigravidae with PM (PM+) at delivery who had normal birth weight (NBW) or LBW infants, and compared them to plasma levels from primigravidae who were PM-negative (PM-) and delivered NBW or LBW infants (Table 2).

**Figure 7:**
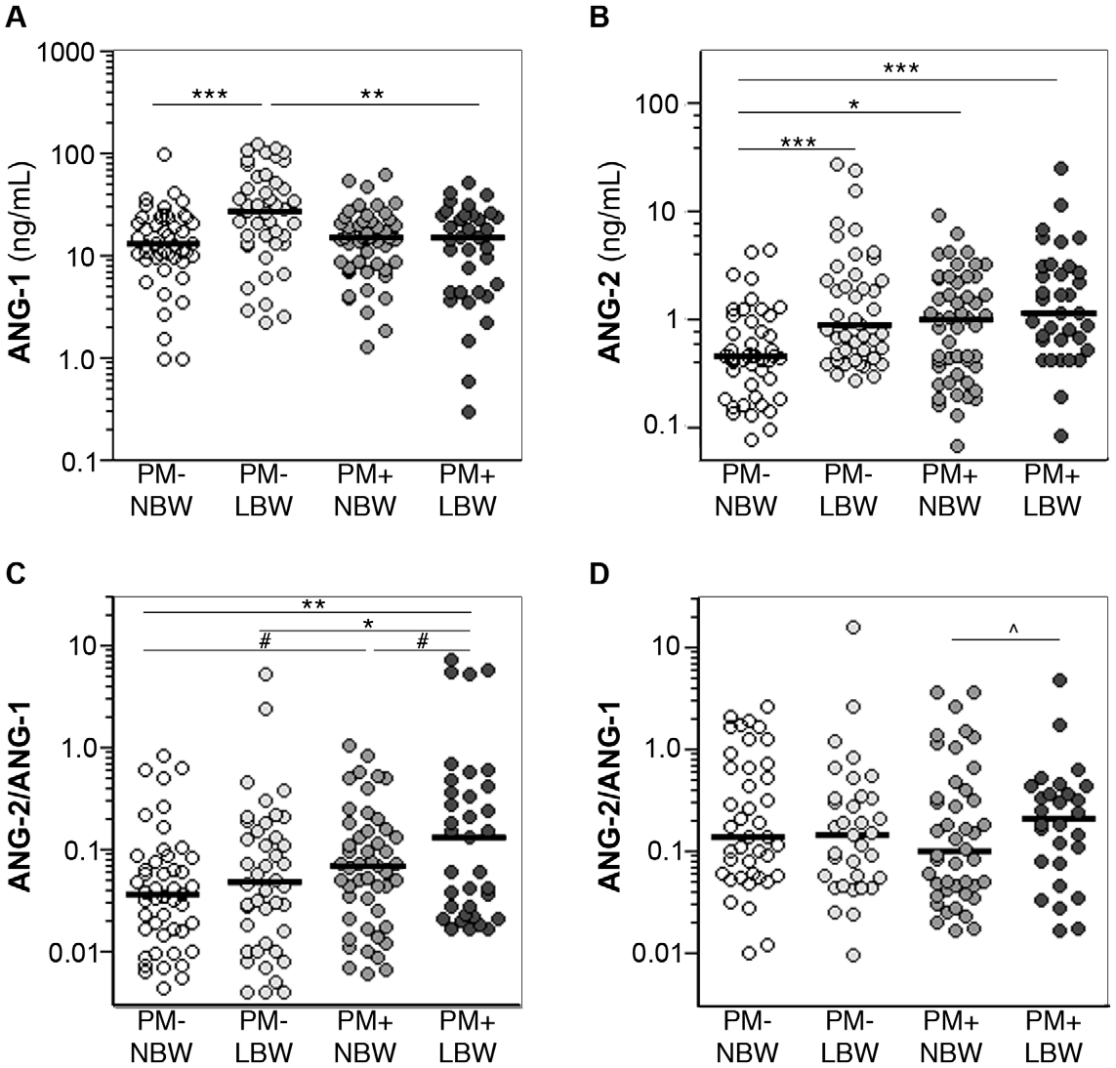
Plasma angiopoietin levels at delivery are dysregulated in PM and with LBW outcomes. *(A–C)* Peripheral plasma and *(D)* matched placental plasma obtained at delivery of normal birth weight (NBW) or LBW infants from primigravid women with (PM+) or without (PM-) PM were measured for ANG-1 and ANG-2 by ELISA. *(A)* Mean maternal peripheral plasma ANG-1 is elevated with LBW deliveries in PM- but not PM+ women. Statistical analyses by Mann-Whitney test. *(B)* Maternal peripheral plasma ANG-2 is elevated with PM. Statistical analyses by Mann-Whitney test. *(C)* Elevated maternal peripheral plasma ANG-2/ANG-1 ratio at delivery is associated with PM (*P* = 0.0016) and LBW (*P* = 0.0406); 2-way ANOVA on log-transformed data. Statistical analyses between groups by t-test on log-transformed data. *(D)* Placental plasma ANG-2/ANG-1 ratio levels are elevated compared to peripheral plasma levels. Statistical analysis by t-test on log-transformed data. Dots represent individual women, lines represent the median of each group. Peripheral plasma, PM- NBW (n = 47), PM- LBW (n = 44), PM+ NBW (n = 51), PM+ LBW (n = 35). Placental plasma, PM- NBW (n = 41), PM- LBW (n = 34), PM+ NBW (n = 44), PM+ LBW (n = 28). * *P*<0.05, ** *P*<0.01, *** *P*<0.001, # *P* = 0.06, ^∧^ *P* = 0.486.

Peripheral ANG-1 levels were similar in all groups of women with the exception of the PM- women who delivered LBW infants. Consistent with the decreased ANG-1 found in association with malaria infection in the experimental PM model and the prospective cohort, PM+ women who delivered LBW infants had decreased peripheral ANG-1 levels compared to PM- women who delivered LBW infants (*P* = 0.002; Fig. 7A).

PM was associated with elevated maternal peripheral ANG-2 levels in primigravid women at delivery of both NBW (*P* = 0.0171) and LBW (*P*<0.0001) infants as compared to the PM- NBW group (Fig. 7B). The placental plasma ANG-2 levels were 2-9 times higher than matched peripheral ANG-2 levels (data not shown), suggesting that the source of systemic angiopoietin levels is the placenta. PM- women who delivered LBW infants also showed an increase in ANG-2, however, as they also had increased ANG-1 levels, their ANG-2:ANG-1 ratio was only modestly higher in this group as compared to the PM- NBW group (Fig. 7C).

When the four groups were analyzed together by 2-way ANOVA, the ANG-2:ANG-1 ratio was significantly increased by both PM (*P* = 0.002) and LBW outcomes (*P* = 0.041). A significant increase in the ANG-2:ANG-1 ratio was found in the peripheral plasma of women in the PM+ LBW group as compared to both the PM- NBW (*P* = 0.002) and PM- LBW (*P* = 0.017). In support of the hypothesis that both PM and LBW are associated with increased angiopoietin dysregulation, the ANG-2:ANG-1 ratio showed a trend towards being higher in the PM+ women at delivery of LBW infants as compared to NBW infants. However, this difference was just beyond statistical significance (*P* = 0.063), as was the increase between the PM- NBW and PM+ NBW groups (*P* = 0.056). A similar trend was observed in the ANG-2:ANG-1 ratio of placental plasma samples (Fig. 7D).

A proportion of the LBW infants were delivered prior to 37 weeks of gestation (i.e., preterm delivery; Table 2). However, no difference in the maternal peripheral plasma ANG-2:ANG-1 levels was observed when stratified based on gestational age at delivery (<37 vs ≥37 weeks; *P* = 0.424, Mann-Whitney test). Furthermore, a lack of correlation between the ANG-2:ANG-1 ratio at delivery and the length of gestation (Spearman coefficient = -0.1672, *P* = 0.1666) suggests the changes in maternal angiopoietin levels at delivery of LBW infants we observed in this cohort (Fig. 7C) cannot be fully explained by premature delivery.

Previous reports have shown an age effect with respect to host response to PM [22]; however, there was no significant difference between angiopoietin levels when stratified by maternal age (≤20 vs >20 years; ANG-1, *P* = 0. 298; ANG-2, *P* = 0.289; ratio, *P* = 0.143).

Taken together, these results show that the relative expression of ANG-1 and ANG-2 at delivery is altered in the context of PM, and that more marked dysregulation of angiopoietins is associated with LBW outcomes.

## Discussion

This study provides the first evidence associating dysregulation of angiopoietins with PM and LBW outcomes. With the objective of providing a plausible mechanistic link between PM and LBW, we examined the expression of the angiogenic factors ANG-1 and ANG-2 in a mouse PM model as well as in two cohorts of malaria-exposed pregnant women. ANG-1 and its antagonist, ANG-2, interact with the same receptor, Tie-2, to regulate endothelial activation and vascular remodeling. Their relative levels provide an indication of the state of endothelial quiescence or activation, with an elevated ANG-2:ANG-1 ratio reflecting active remodeling of the endothelium [35], [36]. This study provides evidence of increased ANG-2:ANG-1 ratio with PM infection, and shows an association between dysregulated angiopoietins and LBW.

We observed a significant decrease in ANG-1 expression with malaria infection during the course of pregnancy in both the mouse model and pregnant women (Figs. 3A, 4, 5, 7A). Similarly, a significant increase in ANG-2 was observed in both the mouse PM model (Figs. 2B, 3B) and in the primigravid women with PM at delivery (Fig. 7B). Elevation of the ANG-2:ANG-1 ratio with PM was also consistently observed (Figs. 2C, 7C). Observed differences in individual angiopoietin levels between the human study cohorts likely reflect differing study designs (prospective vs. cross-sectional) and the dynamic expression levels of angiopoietins during gestation. Our results support the hypothesis that angiopoietin dysregulation is associated with PM, and that more marked angiopoietin dysregulation is associated with LBW outcomes.

Our findings that dysregulated angiopoietin levels are associated with poor pregnancy outcomes are supported by similar findings in preeclampsia, a pathological pregnancy state also associated with IUGR and preterm delivery to which PM has been compared [37]: An increased ratio of systemic ANG-2:ANG-1 in the second trimester has been associated with the development of preeclampsia [12].

Studies using Doppler ultrasound have shown that placental blood flow is altered with malarial infection during pregnancy, and that abnormal uterine artery flow velocity waveforms can develop even late in pregnancy [38], [39]. A decline of ANG-2 in the later part of pregnancy is important to facilitate the switch from branching angiogenesis, which is mediated by synergism of ANG-2 and VEGF [40], to nonbranching angiogenesis, which is required for efficient nutrient and gas exchange [17] and would affect placental blood flow. A failure to make this switch has been shown to be associated with IUGR [41]. Thus, an ANG-2 level that is either too high or sustained too late in gestation could result in formation of a placental vasculature that is inefficient in supporting the full growth potential of the fetus. The elevated ANG-2 levels we observed in primigravid PM+ women supports this hypothesis (Fig. 7B), whereby sustained elevated ANG-2 levels persisting late in gestation could contribute to sprouting of more vessels rather than elongation and terminal differentiation of existing ones. In agreement with these findings, the placental vascular architecture has been reported to have more numerous fetal blood vessels with malaria infection [42]. Histopathology on placentas from the experimental PM model has also been reported to show vascular abnormalities [31].

Given the roles already ascribed to ANG-2, and the known mechanisms associated with PM, it is tempting to hypothesize that ANG-2 is a key mediator of pathology in PM. Placental parasitized erythrocytes are known to bind chondroitin sulfate A expressed on the syncytiotrophoblast, thus sequestering in the intervillous space [43], [44]. Products from burst parasitized erythrocytes within the intervillous space can stimulate macrophages and the syncytiotrophoblast to generate C5a, TNF and other inflammatory cytokines and chemokines [45]-[48], which in turn contribute to the macrophage infiltration associated with LBW outcomes of PM [49]. In such an inflammatory milieu, activated syncytiotrophoblast and macrophages could secrete ANG-2, as immunhistochemical studies have shown both cell types express ANG-2 [13], [18]. Inappropriately timed stimulation of endothelial cells to proliferate and migrate may then prevent appropriate vascular formation required to support the developing fetus.

ANG-2 has also been shown to sensitize endothelium to the effects of TNF [50]. TNF is one of the few factors associated with PM that has been shown to mediate pathology. An increase of TNF in placental plasma of PM+ women has been shown in several studies [42], [51]–[53], and the role of TNF as a mediator of fetal loss associated with placental malaria has been directly demonstrated by TNF-blocking experiments in *Plasmodium*-infected pregnant mice [54], [55] and non-human primates [56]. An increased ANG-2 expression in response to malaria infection may explain in part why pregnant mice infected with low dose of *P. vinkei* required an injection of only 1.5 µg recombinant human TNF to induce fetal loss, while uninfected pregnant mice could withstand 50 µg TNF without any fetal loss [55]. It will be informative in future experiments to test TNF in parallel with ANG-2 and ANG-1, and, in the mouse model, determine if the effect of elevated TNF is abrogated in the absence of elevated ANG-2.

The *P. berghei*/BALB/c model of PM used in this study recapitulates the severe clinical outcomes that make human PM a global health priority: spontaneous abortion and fetal growth restriction, as reproduced in this study (Fig. 1), and preterm delivery [31]. Using such a model offers several advantages for more precise mapping of the complex host-parasite interaction that mediates adverse pregnancy outcomes in PM. These include the ability to control the timing of infection, the inoculum, the timing of data collection, and the opportunity to dissect the contribution of genetic determinants to outcome using modified mouse strains and immunological interventions. Collectively these make the mouse PM model a powerful tool to help understand the pathogenesis of PM, particularly if the findings in the PM model can be linked to clinically relevant outcomes, as we have shown in this study with respect to angiopoietin dysregulation and LBW outcomes.

A limitation of this study is the large proportion of LBW outcomes in the cross-sectional study that were attributable to preterm deliveries (<37 weeks; Table 2). Preterm delivery and IUGR have been suggested to be associated with acute and chronic placental malaria infections, respectively [57]. However, both preterm delivery and IUGR can result from placental insufficiency and inadequate blood flow delivery to the fetus [5]–[7]. In support of preterm delivery and IUGR being defined pathologies along a continuum of poor fetal growth, IUGR has been associated with a higher risk of spontaneous preterm delivery [8], [58]. We did not observe any significant difference in the maternal angiopoietin levels at delivery between women delivering preterm LBW infants and those delivering LBW infants at full term. As such, PM-induced angiopoietin dysregulation may be informative for differentiating LBW infants regardless of etiology. This could have significant clinical relevance as LBW itself is associated with increased infant mortality regardless of gestational age [59].

Based on this study, we cannot exclude the possibility that angiopoietin dysregulation is a consequence of PM but not a cause of fetal growth restriction. Further studies in the mouse PM model involving blocking of ANG-2 or supplementing with ANG-1 will be required to prove causality. Nonetheless, our results suggest that ANG-2:ANG-1 ratio may be informative as a biomarker in screening for those at risk for LBW outcomes. Detection of PM infection is difficult when relying solely on peripheral blood smears due to the propensity of parasites to sequester in the placental intervillous space. Currently, there is no way to distinguish those infected pregnant women at risk of poor fetal outcomes. Thus, a reliable biomarker of PM would be invaluable. Larger prospective studies are required to determine whether malaria-associated angiopoietin dysregulation could predict a LBW outcome, if certain points in gestation are more susceptible to infection-mediated angiopoietin dysregulation, and if dysregulation at different points in gestation lead to different outcomes (i.e., spontaneous abortion vs. preterm delivery vs. IUGR).
